# Making Sure That Orphan Incentives Tip the Right Way in Europe

**DOI:** 10.3390/healthcare10091600

**Published:** 2022-08-23

**Authors:** Denis Horgan, Jasmina Koeva-Balabanova, Ettore Capoluongo, Beata Jagielska, Ivana Cattaneo, Marta Kozaric, Birute Tumiene, Jean-Paul El Ahl, Jonathan A. Lal, Dipak Kalra, Núria Malats

**Affiliations:** 1 European Alliance for Personalised Medicine, 1040 Brussels, Belgium; 2 Department of Molecular and Cellular Engineering, Jacob Institute of Biotechnology and Bioengineering, Faculty of Engineering and Technology, Sam Higginbottom University of Agriculture, Technology and Sciences, Prayagraj 211007, India; 3 Bulgarian Alliance for Precision and Personalized Medicine—BAPPM, 1618 Sofia, Bulgaria; 4 Department of Molecular Medicine and Medical Biotechnologies, University of Naples Federico II, 80131 Naples, Italy; 5 Department of Clinical Pathology and Genomics, Azienda Ospedaliera per l’Emergenza Cannizzaro, 95126 Catania, Italy; 6 Maria Skłodowska-Curie National Research Institute of Oncology, 00-001 Warszawa, Poland; 7 Novartis Farma SpA, 21040 Origgio, Italy; 8 Vilnius University Hospital Santariskiu Klinikos, 08661 Vilnius, Lithuania; 9 Merck nv/sa, 1040 Brussels, Belgium; 10 Department of Genetics and Cell Biology, Institute for Public Health Genomics, GROW School of Oncology and Developmental Biology, Faculty of Health, Medicine and Life Sciences, Maastricht University, 6211 LK Maastricht, The Netherlands; 11 Department of Medical Informatics and Statistics, University of Ghent, 9000 Ghent, Belgium; 12 Spanish National Cancer Research Centre (CNIO), 28029 Madrid, Spain

**Keywords:** orphan medicines, drug development, pharmaceutical strategy, policy framework, regulation, rare diseases, patients, EU regulation, incentives

## Abstract

The delicate balance of funding research and development of treatments for rare disease is only imperfectly achieved in Europe, and even the current provisional equilibrium is under a new threat from well-intentioned policy changes now in prospect that could—in addition to the intrinsic complexities of research—reduce the incentives on which commercial activity in this area is dependent. The European Union review of its pharmaceutical legislation, for which proposals are scheduled to appear before the end of 2022, envisages adjusting the decade-old incentives to meet objectives that are more precisely targeted. However, researchers, physicians, patients and industry have expressed concerns that ill-considered modifications could have unintended consequences in disrupting the current balance and could reduce rather than increase the flow of innovative treatments for rare diseases.

## 1. Introduction

2022 could be a tipping point for the future of orphan drug development. It is an established policy in Europe that “patients suffering from rare conditions should be entitled to the same quality of treatment as other patients,” as the EU Regulation (EC) No. 141/2000 on orphan medicinal products states [1,2,3]. The consensus in force among political authorities is shared with patients, regulators, and the industry that develops orphan medicines. There is consensus too—as the regulation also says—that it is “necessary to stimulate the research, development and bringing to the market of appropriate medications by the pharmaceutical industry” [3]. Right now, debate is flourishing about how these laudable goals can best be achieved in a new decade. The EU is reflecting on how to fine-tune the incentives that provide the stimulus: its Pharmaceutical Strategy explicitly aims to propose before the end of 2022 revisions to the legislation “through more tailored incentives” [4]. The outcome of this debate—and of the subsequent legislative revision—will determine the prospects for many of the 3 million rare-disease patients across Europe who are still in desperate need of treatments [5,6].

There is no doubt that the therapeutic landscape has already been dramatically improved by the 2000 regulation. The investments and efforts it incentivised with its EU-level mechanisms for orphan drug designation, and the accompanying benefits of some marketing exclusivity and reduced fees, have helped the emergence of effective treatments for rare diseases ranging from haemophilia to Gaucher, and from rare forms of cancer, pulmonary hypertension or neonatal diabetes to paroxysmal nocturnal haemoglobinuria—as many patients and their carers can testify. Over the last two decades, rare disease research has resulted in the grant of orphan status to some 2000 products, and the approval of more than 150 orphan drugs—compared with just eight therapies for rare diseases available before the adoption of the regulation [7,8,9]. At least 577 (current and pipeline) rare disease products use new technologies such as cell and gene therapies, antisense RNA interference therapy and monoclonal antibodies to precisely target the disease site [10]. In fact, a survey of approximately 180 expert physicians aimed to identify the most transformative medicines of the past 25 years; shows that of the 26 medicines identified, 10 were first developed for rare diseases [11]. Under the incentive scheme set up by the 2000 regulation, more than 2300 new orphan designations have led to over 190 authorised new treatments [12,13].

However, the successes to date should not be allowed to mask the gaping unmet need across the 6000 conditions currently classified as rare diseases [14,15,16] and among the hundreds of thousands of patients for whom the further development of orphan medicines is the principal hope. The positive trend of the last 20 years cannot be taken for granted. Orphan drug development is difficult, expensive, risky, and with a greater degree of uncertainty than for treatments for more common diseases. As has been well-documented [17], the organisation and implementation of clinical trials for orphan drugs (OMPs) faces difficulties over enrolment of sufficient patients, questionable conclusions from some trials with complex patient populations prone to variability, and ethical questions over the use of placebo. Furthermore, interpretation can be complicated by the heterogeneous and unpredictable presentation of rare diseases, while clinically relevant efficacy can often only be seen after years. In addition, the record on production of evidence of benefits from post-market studies is still uneven. There is another complication to the discussion, in that some authors have suggested that the incentive schemes provide only marginal benefits to patients and society and disproportionate financial advantages to companies [18].

Nonetheless, the EU’s own pharmaceutical strategy highlights the need for a review of the system to improve the delivery of treatments that respond to unmet need, and it recognises explicitly that some form of incentivisation or compensation is appropriate for medicines for rare diseases that pose challenges, both in terms of science and manufacturing [4,19,20,21,22]. The customary difficulties in medicines research impose tougher choices for companies working on rare diseases: on all those points where go/no go decisions have to be made on a development project, the questions are more demanding in rare diseases, the answers less easily predicted, and the balance of probability weighted more towards abandonment [23,24]. The EU regulation was created precisely to compensate for this inherent disadvantage. However, even with that support, a third of the candidate medicines that received formal orphan status in Europe did not make it to patients, because they were abandoned during development, the application was withdrawn, or marketing authorisation was refused. Additionally, of those that did obtain a marketing authorisation, it is estimated that less than 10% are actually marketed [25,26,27]. Since the process of developing a drug for a specific disease, whether it is a rare disease or not, is mostly done in-house, and the process itself is expensive and risky, companies very often choose to develop therapies with the greatest promise of a good financial return. As a result, potential therapies for rare diseases have often faltered, even with the encouragement of the Orphan Drug Act. For product development as well as basic research, a stronger infrastructure is crucial. There is a great need for innovative collaborative strategies to share and leverage resources to reduce research and development (R&D) costs without sacrificing product safety or efficacy. The priority should be to expand resources and capabilities in the preclinical phase of drug development [28]. The fact is that a smaller drug market typically attracts less interest from the pharmaceutical industry, especially in the context of therapeutic development for one disease at a time. Because of that, umbrella-type rare disease organisations can play an important role in the development of rare disease therapeutics [29,30].

To add to the challenges of developers of orphan medicines, they must surmount not just the European regulatory hurdles of winning orphan designation and product approval but must also then find their way through a maze of differing national conditions [31]. These provisions are so different that, as the Pharmaceutical Strategy again acknowledges [4], companies may decide not to market their medicines in one or more countries due to factors such as national pricing and reimbursement policies, size of the population, the organisation of health systems, and national administrative procedures. Studies underline the negative impact of different regulatory frameworks for the development of orphan drugs, with separate governance, multiple assessments, and varying approaches and priorities to unmet medical needs in the public health systems [24]. The challenges are intensified by different value assessment frameworks from country to country, often with a focus on short-term budgetary considerations or simple cost-effectiveness methods. Germany’s relatively congenial route for orphan drugs, directly linking value assessment to significant benefit and establishing a carve-out for orphan products, contrasts with the UK’s less successful approach in which its highly specialised technologies pathway still employs cost-effectiveness thresholds, consequently disqualifying many orphan drugs [1,32].

This diversity is demonstrable simply in comparing across the member states provisions and the specific benefits—where they exist—for orphan drugs: in Austria, Finland, Hungary, Ireland, Latvia, Luxembourg, and Sweden there are no national benefits available. Additionally, in Austria, the reimbursement authority even makes it harder for orphan drugs to prove a patient benefit, because it interprets as a lack of evidence any deficiencies in endpoint data due to fast-track approvals or studies that have been cancelled for ethical reasons [33]. In Italy, the simple molecular testing for some conditions, such as cancer, is not completely implemented in the twenty regions, while the access to many molecular tests is limited by budget availability and the regional rules working in each regional health system [34]. The implementation of Next- Generation Sequencing (NGS) based extended assays for rare disorders (such as whole exome or whole genome sequencing) is really challenging considering that the infrastructure for high-throughput molecular testing is heterogeneous and financial restrictions limit the access to patients needing early diagnosis and, when available, early treatment [35].

## 2. National Perspectives

Table 1 provides a summary of the wide variation in national circumstances relating to the designation and incentives available for developing orphan medicines. Fuller explanations follow in the text.

Among the benefits, orphan medicines qualify for exemption from the national tax on pharmaceuticals and clawbacks in Belgium, and pricing and reimbursement is faster [36]. Bulgaria provides some public funding for orphan drugs [37], and in Croatia very expensive medicines, including orphans, are financed from a dedicated fund [38]. Cyprus includes orphan drugs in its named patient supply mechanism.

The Czech Republic waives administrative, regulatory, and scientific fees, and Denmark offers some waiver of annual fees. France exempts orphan medicines from sales tax, some turnover and other taxes, and from fees on scientific advice. Greece offers a faster and easier pricing and reimbursement procedure. Hungary operates a named patient basis for funding of orphans, but the procedure is very administrative and time consuming. There is no separate reimbursement pathway in Ireland for orphan medicines, and the usual templates are judged by industry to be not fit for purpose [32]. 

Italian legislation has always protected the experimentation of orphan drugs and their entry into the market to guarantee patients access to the best therapies available. Italy provides some tax breaks, exemption from clawbacks, and fee reductions, along with a faster pricing and reimbursement procedure and a dedicated fund for unauthorised orphan drugs awaiting approval. In particular, Law no. 648/1996 allows the supply of some drugs, paid for by the Health National System, to promptly respond to pathological conditions [39]. Finally, Law 326/2003, Art. 48 [40] constituted the AIFA Fund, powered by 5% of the annual expenditure incurred by pharmaceutical companies for promotional activities: 50% dedicated to the purchase of orphan drugs for rare diseases and drugs not yet authorised, but which represent a hope of cure for severe pathologies; the remaining 50% of the fund is for independent drug research. 

In Latvia, only some orphans are reimbursed, while Luxembourg allots prices in line with those of the country of origin. Malta offers 70% fee reductions for academic trials, and the Netherlands possible tax reductions for R&D by high-tech start-ups, as well as some fee waivers and reductions. Poland offers theoretically faster pricing and reimbursement for drugs categorised as innovative drugs. In Portugal orphan drugs are taxed only at 5% and a special use authorisation procedure provides access to some of them. Romania prioritises assessment of pricing dossiers for orphans, Slovakia and Slovenia offer easier reimbursement, and Spain imposes a lower mandatory rebate on orphan drugs [32]. 

## 3. Challenges and Opportunities

However, the very diversity among these benefits (and limitations) demands intricate study by drug companies as they construct their development strategies, which can compound the precarity of the case for work on orphan drugs. Already, the pathway is littered with obstacles: lack of disease knowledge, novel or unproven surrogate endpoints, and small and highly heterogeneous patient populations—all of which impact R&D time and amplify the risk of trial failure compared to more common, better understood conditions. In addition, small patient populations limit the revenue potential of products that successfully reach the market [41,42,43]. There is an important and inescapable economic dimension to the discussion of significance both to society in general, which ultimately carries the costs for most of these—often expensive—products, and for the companies developing them. The discussions of value for society continue to be widely explored—and widely divergent in their findings—and are noted here in passing, although they are not the principal focus of this paper. Anecdotal evidence is regularly produced on both sides of this debate, on the costs of research, the significance of commercial performance by successful innovation in rare-disease treatments, and the merits and costs of more personalised medicine [44,45]. 

However, the impact of the numerous challenges for developers on economic viability—as outlined above are inevitably a major consideration of companies evaluating their investment priorities. Manufacturers compare investment propositions across products and disease areas, and investors weigh options across industries. Investment in the orphan space remains a marginal economic decision in most cases; the economic case for investment, is on average, weak, even in the presence of the incentives in the current regulation. For investment to continue, the risk-adjusted return needs to be commensurate with that from other types of medicines or alternative investment. As a result, legislative provisions have been created aimed at mitigating the market failures linked to low-prevalence, high unmet need conditions [46], in a study commissioned by EFPIA; but echoing observations in the study by Nestler-Parr et al. [47]. Therefore, even the European Society of Paediatric Oncology has stressed the need for “a high level of market protection to facilitate company investment” [1].

Revision of the incentives for orphan drugs will balance competing objectives if it is to be successful. The EU must of course take account of the ambition to maintain economically sustainable health systems and respond to the many concerns over high prices for many orphan drugs [48]. At the same time, it must look squarely at the realities of industrial imperatives: a reflex tightening of the provisions in the regulation for stimulating orphan drug development will risk depriving Europe and European patients of solutions to rare diseases. Recent studies suggest that the calculations employed by the EU—in the Technopolis report on the orphan drug stimulus scheme—do not sufficiently recognise the importance of the incentives [46]. A business economic approach, rather than the Technopolis statistical analysis, suggests that more than half the orphans developed in 2000–2017 would not have been economically viable in the absence of the regulation. The current incentive framework changed the calculus, suggesting that it is well-calibrated to promote the innovation that has been witnessed over the past 20 years. The business economic approach also considered a ‘No Regulation’ scenario and concluded that fewer than half the products would have been developed in those circumstances, because of a lack of economic viability. That would have meant 2 million patients would not have benefited from access to therapy, and Europe would have experienced a EUR 6 billion drop in R&D expenditure [46]. On the other hand, it must be taken into account that every drug, regardless of approval by regulatory medical agencies, in addition to the benefits it should bring, also comes with a potential risk for patients. In the case of rare diseases, even more importance must be attached to this. Given the small number of patients, well-designed clinical trials to determine efficacy can be difficult to conduct, and studies that are large enough to detect serious harms in all cases are nearly impossible and very difficult to conduct. If other treatments are not available, patients may be willing to risk harm for potential benefit, but the benefit-to-harm ratio cannot be easily calculated. If a new drug is unlikely to be widely used, pharmaceutical companies can expect limited financial rewards unless prices are high, in which case the treatment may not be cost-effective [49,50,51]. Hopes of improvements in the EU scheme were boosted in November 2021, when health commissioner Stella Kyriakides told the European Parliament during a debate on rare diseases that the orphan drugs rule has stimulated research and development of medicines for rare diseases and could do more. “More needs to be done since 95% of rare diseases still do not have treatment and orphan medicines are not accessible to patients equally in all member states,” she said [52].

However, unofficial indications that emerged in early 2022 of EU thinking on the changes now planned suggest that vital elements in the current incentive schemes could be drastically impaired—in terms both of eligibility for the scheme and of conditions attached to the benefits it confers. These rumoured tightenings of the calculations of disease prevalence for qualifying, on the duration of a designation, or on the length of market exclusivity granted to authorised orphans are seen by defenders of orphan medicines as serious potential disruptors of the delicate economics of investment in development.

## 4. Discussion

The central conclusion is that in a competitive context for choice of what and where to support financially, a causal relationship exists between incentives and investment. There can be no presumption of R&D in rare diseases, because it is intrinsically uneconomic [53]. The EU incentives have shifted the judgement of viability from insufficient to no more than adequate. Any modifications to the incentives should therefore be made with that in mind. The regulation defines the orphan designation, opening to EU-level advantage, and each country defines the national conditions and potential advantages to facilitate the availability of orphan drugs for patients [24,54,55]. The future definition of orphan drugs, orphan designations criteria, and orphan benefits is crucial both for its EU-level impact, and because it is the key to accessing any associated national-level advantages [53]. Designating a drug as ultra-rare should allow access to national benefits. Narrowing this definition or limiting it to “ultra-rare” diseases by reducing the maximum prevalence allowed would reduce access to EU and national benefits that can make these innovation projects financially viable. This is all the more important since each country can (and regularly does) review the specificities and advantages for orphan drugs. Defending this status at EU level is a minimum, but even then will never guarantee that economic viability of future projects for rare diseases will remain in all countries [56].

Some of the mechanisms in the regulation are under more threat than others. In particular, there is a belief among some policy makers that companies are unduly exploiting the provisions covering multiple indications, notably in the field of oncology, and they seek to limit the corresponding market exclusivity. Under current rules, when an innovation in a rare disease is discovered also to be efficient in another rare disease, it keeps its orphan designation when the second indication is also approved for that other rare disease [25]. This has been characterised as “salami slicing” by some critics, who see it as an artificial device to justify retaining orphan status uniquely for commercial reasons to keep this. The European Commission understandably wishes to calibrate the orphan regulation to encourage research that generates the most value to rare disease patients [57]. However, current thinking on this topic seems to oversimplify the nature of unmet need and misunderstand the way that innovation incrementally reduces it. As a single medicine almost never fully alleviates a disease burden, restricting the definition of innovative only to first-in-disease drugs (and thus also limiting the eligibility to incentives) is a mistake. Multiple waves of innovation will be necessary to meaningfully reduce disease burden. Disincentivising follow-on medicines will impede attainment of the goal. Accusations of salami-slicing ignore the evolutionary nature of medical science and reject the movement towards personalisation of medicine, based on constant improvements in the understanding of cancers, in identifying more precisely the different types, and distinguishing them with different criteria, for example via a biomarker. The claim that an OMP does not merit incentivisation because there is already a treatment for that condition ignores the obvious reality that the existing treatment may not be entirely effective or well-tolerated or easily administered. A blanket exclusion from OMP status on such arbitrary grounds’ risks compounding rather than resolving the continuing challenge of massive unmet need. Similarly, the charge that companies abusively focus on familiar and well-served therapeutic categories may misread the underlying process of research, which is not susceptible to the form of advance planning that can designate years in advance what any particular avenue of investigation will deliver.

The argument is made by critics of the incentives scheme that the industry is being disingenuous when warning that lower prices or lack of incentives are linked to delayed launch in particular countries or regions. There is evidence, however, from industry-commissioned studies that the pattern of delayed launch is consistently observed in countries and regions with low prices and barriers to reimbursement. The average delay between market authorisation and patient availability for orphan drugs can be as short as 3.5 months in some countries or as long as 3.6 years—and the countries that suffer the greatest delay are typically in eastern Europe and the Baltics. In total, 25% of countries studies do not have availability to any non-oncology orphan drugs approved in 2020, and 30% of countries studied have availability to less than 10% of the non-oncology orphan drugs approved between 2017 and 2020 [58]. The complexities of product approval and launch in general are also extensively discussed in the article, which shows that it takes an average of 511 days for innovative treatments to be granted reimbursement in Europe, with a range that varies from 133 days in Germany to 899 days in Romania.

With the increase in the number of approved drugs for rare diseases, the high prices associated with some of the medicines have come into focus of discussion. In a detailed study published by Jayasundara et al., comparing the differences in clinical costs between orphan and non-orphan drugs, they found that out-of-pocket clinical costs per approved orphan drug were USD 166 million, while for a non-orphan drug they were USD 291 million. Moreover, the capitalised clinical costs are also lower for approved orphan drugs than for non-orphan drugs. It may be that the high prices of drugs for rare diseases allow drug R&D and manufacturing costs to be reimbursed from a relatively small number of patients. For both groups of drugs, out-of-pocket costs increase with the clinical trial phase, and therefore, according to this whole perspective, healthcare costs could be better regulated, at least on the basis of research and development and production costs to ensure that the patients have access to the best care [21,59,60].

The future of research depends on greater differentiation of pathologies, identification of new sub-pathologies, and the provision of ever better adapted treatments to deliver better clinical outcomes [8,53,61]. In this regard, the latest recommendations by Souche et al. focused on the Horizon2020 project Solve-RD which has the ambition to elucidate the genetic cause of the majority of currently unsolved rare genetic disorders by a variety of analytical techniques [62]. This mean that the harmonisation of diagnostic NGS-based procedures is still challenging worldwide: therefore, together with the regulatory and financial support of the development of orphan drugs, the harmonisation of diagnostic procedures will also have to be supported by EU, in order to implement availability, the of a precision molecular diagnostic with high quality standards.

The benefit of market exclusivity also faces a challenge of enforceability at member state level, and this has the effect of discouraging continued investments to develop and launch new indications for an existing orphan drug [1,63]. The value of a multi-indication approach in rare diseases is that every new indication is a completely new disease, which in most cases, lacks authorised treatments—in contrast to the situation in more prevalent diseases, where companies often expand their indications to enable treatment of less severe patients suffering from the same original condition. This difference extends to the impossibility of making virtually simultaneous multi-indication launches and underlines the inevitability of staggered introduction—since it can take 10–15 years to develop all possible indications.

## 5. Conclusions

The conclusion to be drawn from these observations is that the revision of the incentives in the orphan drug regulation needs to be conducted with caution, and in full understanding of the dynamics influencing drug development. If the EU were to bow to the evident pressures for reduced incentives, it would disrupt what is already a tenuous equilibrium in European development of orphan medicines. The EU might, instead, contemplate promoting a well-informed and broadly accessible discussion of the factors governing orphan drug development. The real risk is not that there will be no more orphan medicines, but there may be no more orphans launched in Europe, because the economic viability of European engagement—with a combination of low prices and insufficient incentives—would be jeopardised. European investment would move away from rare diseases, and the development of orphans would gravitate to regions where incentives and prices offered better compensation for the additional risks of orphan drug development. Additionally, where prices are higher—the US being the most obvious example—and at a time when the trend is inexorably towards international reference pricing, orphan drug developers may hesitate to launch in lower-priced regions for fear of contamination of their higher prices [64].

European society’s goals for equitable access to treatments will be met only if the economic incentives are right, if they provide for the prospect of profit to reward investment decisions and inform go/no go calls as a potential medicine emerges. For orphans, with smaller commercial opportunities, this calculation is still more acute, and market exclusivity and price need to balance the equation. Lower prevalence needs compensation through higher prices and/or compensating incentives. Maintaining a positive incentive framework is essential to advancing therapeutic innovation towards effective preventative medicines and treatments for rare diseases, strengthening equitable health systems, and fostering a productive biopharmaceutical industry in Europe. If the EU is indeed to revise its incentives for orphan drugs, 2022 could be the crucial year for ensuring that it tips the right way.

## Figures and Tables

**Table 1:** Different national perspectives in orphan incentives.

Country	Perspective
Belgium	Orphan medicines qualify for exemption from the national tax on pharmaceuticals and clawbacks; pricing and reimbursement are faster;
Bulgaria	Some public funding is provided for orphan drugs;
Croatia	Orphans are financed from a dedicated fund;
Cyprus	Orphan drugs are included in its named patient supply mechanism;
Czech Republic	No administrative, regulatory and scientific fees;
Denmark	Certain waiver of annual fees is offered;
France	Orphan medicines are exempted from sales tax, some turnover, and other taxes, and from fees on scientific advice;
Greece	A faster and easier pricing and reimbursement procedure is offered;
Hungary	A named patient basis for funding of orphans is operated, but the procedure is very administrative and time consuming;
Ireland	There is no separate reimbursement pathway for orphan medicines, and the usual templates are judged by industry to be not fit for purpose;
Italy	Legislation protects the experimentation of orphan drugs and their entry into the market to guarantee patients access to the best therapies available; some tax breaks are provided, exemption from clawbacks, and fee reductions, along with a faster pricing and reimbursement procedure and a dedicated fund for unauthorised orphan drugs awaiting approval
Latvia	Only some orphans are reimbursed;
Luxembourg	Prices are allotted in line with those of the country of origin;
Malta	70% fee reductions for academic trials are offered
Netherlands	Possible tax reductions for R&D by high-tech start-ups are offered, as well as some fee waivers and reductions;
Poland	Theoretically faster pricing and reimbursement for drugs categorised as innovative drugs are offered;
Portugal	Orphan drugs are taxed only at 5% and a special use authorisation procedure provides access to some of them;
Romania	Assessment of pricing dossiers for orphans is prioritised;
Slovakia	Easier reimbursement for orphan medicines is offered;
Slovenia	Easier reimbursement for orphan medicines is offered;
Spain	Lower mandatory rebate on orphan drugs is imposed.

## Data Availability

Not applicable.
